# Optimal target of LDL cholesterol level for statin treatment: challenges to monotonic relationship with cardiovascular events

**DOI:** 10.1186/s12916-022-02633-5

**Published:** 2022-11-14

**Authors:** Masashi Sakuma, Satoshi Iimuro, Tomohiro Shinozaki, Takeshi Kimura, Yoshihisa Nakagawa, Yukio Ozaki, Hiroshi Iwata, Katsumi Miyauchi, Hiroyuki Daida, Satoru Suwa, Ichiro Sakuma, Yosuke Nishihata, Yasushi Saito, Hisao Ogawa, Masunori Matsuzaki, Yasuo Ohashi, Isao Taguchi, Shigeru Toyoda, Teruo Inoue, Ryozo Nagai

**Affiliations:** 1 Department of Cardiovascular Medicine, Dokkyo Medical University School of Medicine, 880 Kitakobayashi, Mibu, Tochigi, 321-0293 Japan; 2 Innovation and Research Support Center, International University of Health and Welfare, Tokyo, Japan; 3 Department of Information and Computer Technology, Faculty of Engineering, Tokyo University of Science, Tokyo, Japan; 4 Department of Cardiovascular Medicine, Kyoto University Graduate School of Medicine, Kyoto, Japan; 5 Department of Cardiovascular Medicine, Shiga University of Medical Science, Otsu, Japan; 6 Department of Cardiology, Fujita Health University Okazaki Medical Center, Okazaki, Japan; 7 Department of Cardiovascular Medicine, Juntendo University Graduate School of Medicine, Tokyo, Japan; 8 Department of Cardiovascular Medicine, Juntendo University Shizuoka Hospital, Izunokuni, Japan; 9 Caress Sapporo Hokko Memorial Clinic, Sapporo, Japan; 10 Department of Cardiovascular Medicine, St. Lukes International Hospital, Tokyo, Japan; 11 Chiba University, Chiba, Japan; 12 Kumamoto University, Kumamoto, Japan; 13 St. Hill Hospital, Ube, Japan; 14 Department of Integrated Science and Technology for Sustainable Society, Chuo University, Tokyo, Japan; 15 Department of Cardiology, Dokkyo Medical University Saitama Medical Center, Koshigaya, Japan; 16 Japan Red Cross Society, Nasu Red Cross Hospital, Otawara, Japan; 17 Jichi Medical University, Shimotsuke, Japan

**Keywords:** LDL cholesterol, Target value, Threshold value, Statin, Coronary artery disease, Proportional hazard, Bottoming-out model

## Abstract

**Background:**

Aggressive lipid lowering by high-dose statin treatment has been established for the secondary prevention of coronary artery disease (CAD). Regarding the low-density lipoprotein cholesterol (LDL-C) level, however, the “The lower is the better” concept has been controversial to date. We hypothesized that there is an optimal LDL-C level, i.e., a “threshold” value, below which the incidence of cardiovascular events is no longer reduced. We undertook a subanalysis of the REAL-CAD study to explore whether such an optimal target LDL-C level exists by a novel analysis procedure to verify the existence of a monotonic relationship.

**Methods:**

For a total of 11,105 patients with CAD enrolled in the REAL-CAD study, the LDL-C level at 6 months after randomization and 5-year cardiovascular outcomes were assessed. We set the “threshold” value of the LDL-C level under which the hazards were assumed to be constant, by including an artificial covariate max (0, LDL-C − threshold) in the Cox model. The analysis was repeated with different LDL-C thresholds (every 10 mg/dl from 40 to 100 mg/dl) and the model fit was assessed by log-likelihood.

**Results:**

For primary outcomes such as the composite of cardiovascular death, non-fatal myocardial infarction, non-fatal ischemic stroke, and unstable angina requiring emergency hospitalization, the model fit assessed by log-likelihood was best when a threshold LDL-C value of 70 mg/dl was assumed. And in the model with a threshold LDL-C ≥ 70 mg/dl, the hazard ratio was 1.07 (95% confidence interval 1.01–1.13) as the LDL-C increased by 10 mg/dl. Therefore, the risk of cardiovascular events decreased monotonically until the LDL-C level was lowered to 70 mg/dl, but when the level was further reduced, the risk was independent of LDL-C.

**Conclusions:**

Our analysis model suggests that a “threshold” value of LDL-C might exist for the secondary prevention of cardiovascular events in Japanese patients with CAD, and this threshold might be 70 mg/dl for primary composite outcomes.

**Trial registration:**

http://www.clinicaltrials.gov. Unique identifier: NCT01042730.

**Supplementary Information:**

The online version contains supplementary material available at 10.1186/s12916-022-02633-5.

## Background

Epidemiological studies have shown that an elevated low-density lipoprotein cholesterol (LDL-C) level is a major risk factor for cardiovascular events, and it has been established that LDL-C lowering with 3-hydroxy-3-methylglutaryl coenzyme A (HMG-CoA) reductase inhibitors (statins) is effective for the primary and secondary prevention of coronary artery disease (CAD) [1–8]. There has been a growing number of clinical trials for the secondary prevention of CAD, in which aggressive and conventional statin therapies were compared [9–12]. A meta-analysis of these trials found that the odds ratio of aggressive therapy to conventional therapy for the development of cardiovascular events was 0.84 (95% confidence interval: 0.80–0.89) [13]. On the basis of these results, a level below 70 mg/dl was recommended as a goal of LDL-C lowering management in CAD patients [14]. A subanalysis of the Justification for the Use of the Statins in Prevention: an Intervention Trial Evaluating Rosuvastatin (JUPITER) trial showed that the attainment of LDL-C <50 mg/dl achieved a lower risk of cardiovascular events compared with the failure to do so in high-risk CAD patients [15], leading to the “The lower, the better” concept. In addition, recent trials for non-statin lipid-lowering therapy, including ezetimibe and proprotein convertase subtilisin/kexin type 9 (PCSK9) inhibitors, have strengthened the “The even lower, the even better” concept for LDL-C [16–18]. Actually in the clinical setting, however, we occasionally experience cardiovascular events even if LDL-C is extremely lowered by using PCSK9 inhibitors [19]. In addition, the “The lower, the better” concept was based on American and European evidence, and it is uncertain that it can be applied to Japanese and east Asian CAD patients, who have a lower cardiovascular event risk than CAD patients in Western countries. Therefore, we hypothesized that the LDL-C level that should be targeted for Japanese CAD patients to reduce cardiovascular events would be higher than that for Western patients and that there would be an optimal target of LDL-C, i.e., a “threshold” value, below which it does not affect the incidence of cardiovascular events.

Our group conducted the Randomized Evaluation of Aggressive or Moderate Lipid Lowering Therapy With Pitavastatin in Coronary Artery Disease (REAL-CAD) trial, which demonstrated that high-dose (4 mg/day) pitavastatin therapy could achieve a greater reduction of cardiovascular events than low-dose (1 mg/day) pitavastatin in Japanese stable CAD patients [20]. To test our hypothesis, we performed a subanalysis of the REAL-CAD study to explore whether such an optimal LDL-C level exists in Japanese CAD patients.

## Methods

### REAL-CAD trial

The REAL-CAD trial is a prospective, multicenter, randomized, open-label, blinded endpoint trial, to explore whether high-dose pitavastatin (4 mg/day) reduces cardiovascular events in Japanese patients with stable CAD, compared with low-dose pitavastatin (1 mg/day). After a run-in period of at least 1 month on pitavastatin 1 mg/day, eligible patients were randomized to two groups, either receiving an increased dose of 4 mg/day pitavastatin or continuing on 1 mg/day pitavastatin [20].

The study protocol was approved by the Public Health Research Foundation ethics review committee and by the ethics committees at all participating centers. All the study patients provided written informed consent.

### Analysis set

**Fig. 1:**
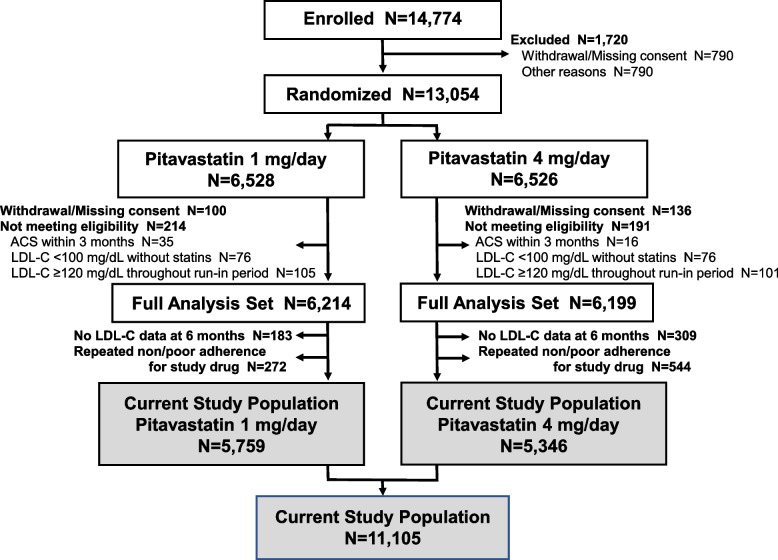
CONSORT diagram for the study population. The full analysis set indicated the modified intention-to-treat population in the main analysis of the trial, while the current study population, indicated per-protocol population for the present substudy, was 11,105 patients (pitavastatin 1 mg/day group: *N*=5779, pitavastatin group: 4 mg/day group: *N*=5346). ACS, acute coronary syndrome; LDL-C, low-density lipoprotein cholesterol; *N*, number

The present study was performed as a post hoc analysis, the plan of which had been reviewed and approved by the REAL-CAD steering committee. In the REAL-CAD trial, 13,054 patients with LDL-C <120 mg/dl on pitavastatin 1 mg/day during the run-in period were randomized to pitavastatin 1 mg/day or 4 mg/day. Among the full set of 12,413 patients in the main study, 11,921 had available 6-month LDL-C data. In these patients, 816 with reported non/poor adherence for the study drug during the first 6 months after randomization were excluded from this subanalysis, based on the agreement of the REAL-CAD steering committee for subanalyses. Therefore, the population for the current subanalysis consisted of 11,105 patients (pitavastatin 1 mg/day: 5759 patients; pitavastatin 4 mg/day: 5346 patients) without reported non-adherence for the study drug (Fig. 1).

### Variables

In the REAL-CAD trial, blood lipid levels (total cholesterol, LDL-C, triglyceride (TG), and high-density lipoprotein cholesterol (HDL-C)), as well as other blood parameters, including creatine kinase, hemoglobin A1c, and indices of hepatic and renal function, were measured at baseline, 6 months, 12 months, and yearly thereafter. High-sensitivity C-reactive protein (hsCRP) was also measured at baseline and at 6 months. LDL-C was calculated by the Friedewald equation using data from the central laboratory. If it was missing, the value was not imputed from other data, but recorded as a missing value.

The variables we used were age; gender; body mass index; current smoking; presence of hypertension, diabetes mellitus, and chronic kidney disease; LDL-C level at baseline and at 6 months; baseline HDL-C and TG; hsCRP at baseline and at 6 months; concomitant medications, such as beta blockers, dual antiplatelet therapy, and angiotensin-converting enzyme (ACE) inhibitors/angiotensin receptor blockers (ARBs); and prior disease history, such as myocardial infarction, unstable angina, percutaneous coronary intervention (PCI), coronary artery bypass surgery (CABG), stroke, atrial fibrillation, malignancy, and chronic heart failure.

### Endpoints

The endpoints were (1) the primary endpoint of the REAL-CAD trial, namely the composite of cardiovascular death, non-fatal myocardial infarction, non-fatal ischemic stroke, and unstable angina requiring emergency hospitalization, and the following secondary endpoints of the REAL-CAD trial: (2) cardiovascular death, (3) myocardial infarction, (4) ischemic stroke, and (5) hemorrhagic stroke. Cardiovascular death consisted of cardiac death, including sudden death and cardiac procedure-related death, as well as noncardiac vascular death. Death without obvious noncardiovascular causes was regarded as cardiovascular death. Myocardial infarction was defined as described by the Academic Research Consortium (ARC). Stroke included both fatal and non-fatal events, excluding procedure-induced ones. Hemorrhagic stroke included both fatal and non-fatal events, excluding procedure-induced ones. The follow-up period for each endpoint was as follows: (1) event occurrence, (2) dropout, or (3) study period, whichever came first [20].

### Statistical analysis

Our purpose was to investigate the association between each endpoint and achieved LDL-C levels. We combined two randomized groups into one and performed the analyses on the total data. Background characteristics were summarized as a percentage, mean and standard deviation, or quartiles, as appropriate. For patients with the primary endpoint, we retrospectively displayed the distributions of LDL-C at every 12-month visit before the event (with the last observation carried forward). Incidence rates per 1000 person-years of each endpoint were summarized by LDL-C level categories and compared by the Cox models adjusting for the following variables: gender; age (≥ 65 or <65 years); obesity (body mass index ≥ 25 or <25 kg/m^2^); current smoking; presence of hypertension, diabetes mellitus, and chronic kidney disease; HDL-C (≥40 or <40 mg/dl); TG (≥ 150 or <150 mg/dl); hsCRP (≥1.0 or <1.0 mg/l); concomitant medications (beta blockers, dual antiplatelet therapy, or ACE inhibitors/ARBs); previous history for myocardial infarction, unstable angina, PCI, CABG, stroke, atrial fibrillation, malignant tumor, and chronic heart failure; and randomized group (1 mg or 4 mg pitavastatin per day). We then fitted the multivariable Cox model including LDL-C level as a continuous variable, which assumed a linear increase in log-hazards with a 1-unit increase in LDL-C. This linearity assumption implicitly reflects the “The lower, the better” concept. As explained in the introduction, however, this concept would be questionable. Therefore, alternatively, we set the “threshold” value of the LDL-C level, below which the hazards were assumed to be constant, by including an artificial covariate max (0, LDL-C − threshold) in the Cox model. We named this model the “bottoming-out model.” The analysis was repeated with the different LDL-C assumed thresholds (every 10 mg/dl from 40 to 100 mg/dl) and the model fit was assessed by log-likelihood. Five-year risks under the fitted Cox models with different thresholds were estimated by standardizing the predicted risks of all patients, calculated from the estimates of baseline-hazard and regression coefficients with LDL-C level set at targeted levels with all the other covariates fixed [21]. We used SAS version 9.4 (Cary, NC) for all analyses.

## Results

We analyzed 5-year outcomes in a total of 11,105 patients. The median follow-up period for study endpoints in the study population was 3.95 years. As a result, the primary composite outcome occurred in 533 patients (5-year risk: 6.68%). In each group of pitavastatin 1 mg/day (5759 patients) and pitavastatin 4 mg/day (5346 patients), the primary composite outcomes occurred in 308 (5-year risk: 7.53%) and 225 (5-year risk: 5.76%) patients, respectively.

Table 1 shows the baseline characteristics of the subjects in the subanalysis. A total of 11,105 patients were aged 68.1±8.3 years and included 82.8% males. In each group of pitavastatin 1 mg/day (5759 patients) and pitavastatin 4 mg/day (5346 patients), age (68.1±8.3 vs. 68.0±8.3 years) and male gender (82.5 vs. 83.2%) were similar. Baseline LDL-C levels at randomization were 87.8±18.9 and 87.7±18.8 mg/dl, and the levels at 6 months were 89.0±21.1 and 72.2±18.8 mg/dl (*p* < 0.0001) in the pitavastatin 1 mg/day and pitavastatin 4 mg/day groups, respectively. There were no significant differences between the 1 mg/day and 4 mg/day groups in the other background data.

**Table 1:** Baseline characteristics

	Total (*n*=11,105)	Pitavastatin 1 mg/day (*n* = 5759)	Pitavastatin 4 mg/day (*n* = 5346)
Age, yr	68.1±8.3	68.1±8.3	68.0±8.3
Age ≥65 yr, *n* (%)	7512 (67.7)	3913 (68.0)	3599 (67.3)
Male gender, *n* (%)	9197 (82.8)	4475 (82.5)	4447 (83.2)
Body mass index, kg/m^2^	24.7±3.4	24.6±3.4	24±3.3
Body mass index <25 kg/m^2^, *n* (%)	6024 (57.8)	3150 (58.3)	2874 (57.4)
Risk factor, *n* (%)			
Current smoking	1812 (16.3)	912 (15.8)	900 (16.8)
Hypertension	8447 (76.1)	4366 (75.8)	4081 (76.3)
Diabetes mellitus	4445 (40.1)	2296 (39.9)	2159 (40.4)
Chronic kidney disease	3918 (35.8)	2055 (36.2)	1863 (35.4)
LDL-C, mg/dl			
Baseline	87.8±18.8	87.8±18.9	87.7±18.8
6 months	81.1±21.6	89.0±21.1	72.7±18.8
Change (6 months − baseline)	−6.6±19.1	1.2±17.4	−15.0±17.1
HDL-C (baseline), mg/dl	50.6±12.4	50.7±12.7	50.5±12.2
Baseline HDL-C <40mg/dl, *n* (%)	1977 (17.8)	1045 (18.2)	932 (17.4)
TG (baseline), mg/dl	124.0 (89.1, 174.0)	124.0 (88.0, 173.0)	125.0 (89.0, 177.0)
Baseline TG ≥150mg/dl, *n* (%)	3913 (35.3)	2002 (34.8)	1911 (35.8)
hsCRP (6 months), mg/l	0.480 (0.226, 1.070)	0.520 (0.245, 1.170)	0.439 (0.207, 0.960)
hsCRP (6 months) ≥1.0 mg/l, *n* (%)	2830 (26.7)	1594 (29.0)	1236 (24.1)
Concomitant medications, *n* (%)			
Beta blockers	4370 (42.1)	2273 (42.2)	2097 (42.1)
Dual antiplatelet therapy	4599 (44.3)	2395 (44.4)	2204 (44.2)
ACE inhibitors/ARBs	7006 (67.5)	3636 (67.5)	3370 (67.6)
Previous history, *n* (%)			
Myocardial infarction	5767 (51.9)	2994 (52.0)	2773 (51.9)
Unstable angina	2799 (25.2)	1451 (25.2)	1348 (25.2)
PCI	9304 (83.8)	4800 (83.4)	4504 (84.3)
CABG	1432 (12.9)	741 (12.9)	691 (12.9)
Stroke	851 (7.7)	454 (7.9)	397 (7.4)
Atrial fibrillation	684 (6.2)	362 (6.3)	322 (6.0)
Malignant tumor	592 (5.3)	328 (5.7)	264 (4.9)
Chronic heart failure	566 (5.1)	309 (5.4)	257 (4.8)

Data are expressed as mean±standard deviation or median and interquartile range for continuous variables and number and percentage for categorical variables. *LDL-C* low-density lipoprotein cholesterol, *HDL-C* high-density lipoprotein cholesterol, *TG* triglyceride, *hsCRP* high-sensitivity C-reactive protein, *ACE* angiotensin-converting enzyme, *ARBs* angiotensin receptor blockers, *PCI* percutaneous coronary intervention, *CABG* coronary artery bypass graft surgery

**Fig. 2:**
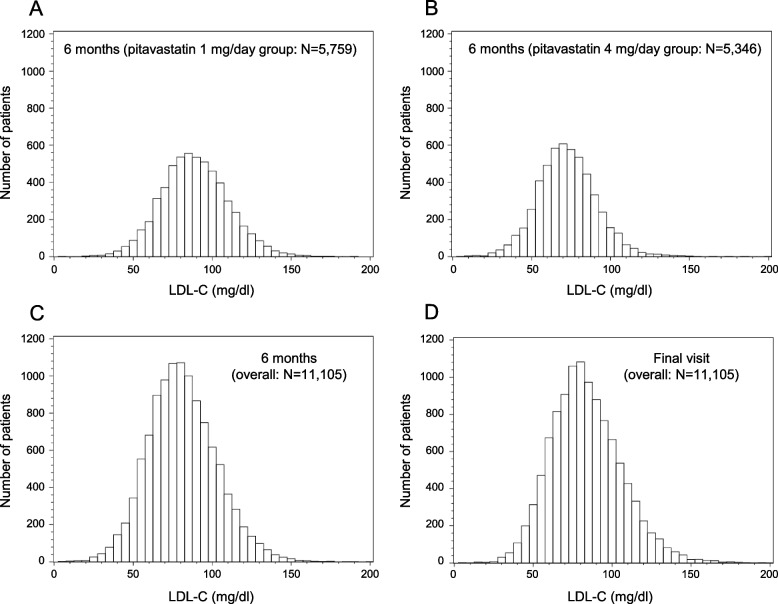
Histograms for distribution of LDL-C levels at 6 months and at the most recent event occurrence. **A** At 6 months in the pitavastatin 1 mg/day group. **B** At 6 months in the pitavastatin 4 mg/day group. **C** At 6 months in overall patients. **D** At the most recent event occurrence in overall patients

**Fig. 3:**
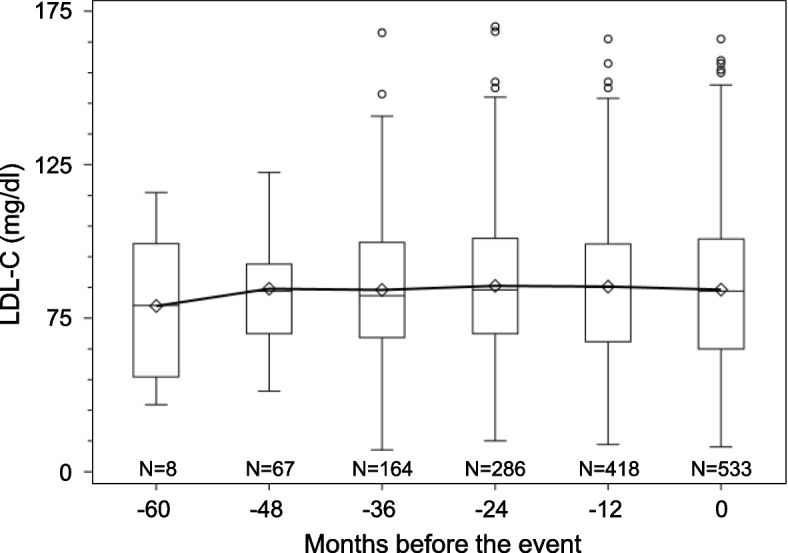
The box and whisker plot of LDL-C at different timepoints before the primary event among the cases (*n* = 533). The means are connected with the line. The box indicates the first and third quartiles and the whiskers are drawn to the minimum or maximum values within the 1.5 interquartile range from the edges of each box

Figure 2 shows the distributions of LDL-C levels. In both the 1 mg/day and 4 mg/day groups, LDL-C was equally distributed on both sides, centered at 89.0 mg/dl and 72.7 mg/dl, respectively. At 6 months, the LDL-C levels were lower in the pitavastatin 4 mg/day group than in the pitavastatin 1 mg/day group (Fig. 2A, B). In the overall patients, the distribution was similar in both groups at 6 months and at the final visit during the follow-up period for the primary endpoint (Fig. 2C, D), indicating that LDL-C control continued well at the final visit as well as at 6 months. Figure 3 shows the serial change in LDL-C retrospectively from just before the event, focusing on the subject who had developed the primary composite events. We can see that the LDL-C control did not deteriorate before the event in these patients.

We divided the 11,105 patients into 6 categories by the LDL-C level at 6 months and calculated event rates and multivariable-adjusted hazard ratios among each category. When the category 100≤ LDL-C <125 mg/dl was used as a reference, based on Japanese guidelines [22] the event rates and adjusted hazard ratios were the lowest in the category of 50 ≤ LDL-C < 75 mg/dl for primary composite outcomes, myocardial infarction and ischemic stroke. In the category of LDL-C < 50 mg/dl, the event rate increased. For cardiovascular death, the event rate was lowest in the category 75 ≤ LDL-C < 100 mg/dl and the hazard ratios were lowest in categories 50 ≤ LDL-C < 75 mg/dl and 75 ≤ LDL-C < 100 mg/dl. No clear trend was observed for hemorrhagic stroke (Table 2). Then, a similar analysis was performed separately in each group of pitavastatin 1 mg/day (*n*=5759) and pitavastatin 4 mg/day (*n*=5346). Consequently, a trend of the result shown in overall patients was more prominently recognized in the pitavastatin 4 mg/day group, but was absent in the pitavastatin 1 mg/day group. However, the number of patients included in category of LDL-C < 50 mg/dl was extremely small in the pitavastatin 1 mg/day group (Additional files 1 and 2: Table S1 and S2).

**Table 2:** LDL-C category-specific event rates

Endpoint	Achieved on-trial LDL-C level (mg/dl)					
	LDL-C < 50	50 ≤ LDL-C < 75	75 ≤ LDL-C < 100	100 ≤ LDL-C < 125	125 ≤ LDL-C < 150	150 ≤ LDL-C
	*n* = 659	*n* = 3831	*n* = 4559	*n* = 1719	*n* = 306	*n* = 31
Primary composite outcome, *n*	32	157	230	97	15	2
Rate (/1000 person-years)	13.1	10.9	13.6	15.3	13.5	18.6
Adjusted HR (95% CI)	0.78 (0.49, 1.22)	0.70 (0.52, 0.93)	0.87 (0.67, 1.13)	Ref	1.01 (0.57, 1.79)	1.11 (0.27, 4.52)
Cardiovascular death, *n*	12	58	64	28	7	1
Rate (/1000 person-years)	4.8	4.0	3.7	4.3	6.2	9.2
Adjusted HR (95% CI)	0.92 (0.42, 2.00)	0.87 (0.52, 1.47)	0.87 (0.53, 1.42)	Ref	1.65 (0.67, 4.08)	1.93 (0.26, 14.44)
Myocardial infarction, *n*	5	25	47	23	3	0
Rate (/1000 person-years)	2.0	1.7	2.7	3.6	2.7	-
Adjusted HR (95% CI)	0.56 (0.18, 1.72)	0.49 (0.25, 0.94)	0.72 (0.41, 1.24)	Ref	0.90 (0.27, 3.05)	-
Ischemic stroke, *n*	10	47	60	21	4	0
Rate (/1000 person-years)	4.1	3.2	3.5	3.3	3.6	-
Adjusted HR (95% CI)	1.12 (0.50, 2.52)	0.89 (0.50, 1.60)	0.98 (0.57, 1.69)	Ref	1.26 (0.43, 3.74)	-
Hemorrhagic stroke, *n*	3	34	13	11	2	0
Rate (/1000 person-years)	1.2	2.3	0.8	1.7	1.8	-
Adjusted HR (95% CI)	0.97 (0.24, 3.86)	1.69 (0.74, 3.87)	0.50 (0.20, 1.26)	Ref	0.77 (0.10, 6.17)	-

Adjusted for gender, age (<65 or 65≤ years), obesity (body mass index <25 or 25≤ kg/m^2^), diabetes mellitus, hsCRP (<1.0 or 1.0≤ mg/dl), TG (<150 or 150≤ mg/dl), HDL-C (<40 or 40≤ mg/dl), drug use (beta blocker, DAPT, or ACEI/ARB), disease history (MI, unstable angina, PCI, CABG, stroke, AF, malignant tumor, chronic heart failure, hypertension, chronic kidney disease), current smoking, and randomized group (pitavastatin 1 mg/day or 4 mg/day). Adjusted HR and 95% CI in each category are shown as the values when the category 100≤ LDL-C <125 was used as a reference

*CI* confidence interval, *HR* hazard ratio, *LDL-C* low-density lipoprotein cholesterol

Next, to explore the optimal target of LDL-C, we assumed a “threshold” value, below which the incidence of cardiovascular events is not further lowered, and then calculated multivariable-adjusted hazard ratios for each of the threshold values set by every 10 mg/dl. For example, when we set the threshold value as 60 mg/dl, the adjusted hazard ratio was calculated after the values <60 mg/dl had been converted to 60 mg/dl. Consequently, for primary composite outcomes, the model fit assessed by log-likelihood was best when the threshold LDL-C value of 70 mg/dl was assumed. And in the model with a threshold LDL-C value of ≥ 70 mg/dl, the hazard ratio was 1.07 (95% confidence interval 1.01–1.13) when LDL-C increased by 10 mg/dl. A similar trend was observed in cardiovascular death, myocardial infarction, and ischemic stroke. The best model fit was observed with an assumed threshold LDL-C of 80 mg/dl for cardiovascular death, 70 mg/dl for myocardial infarction, and 60 mg/dl for ischemic stroke (Table 3).

**Table 3:** Multivariable-adjusted HR estimates for the 10-mg/dl increase in LDL-C with or without threshold

LDL-C (mg/dl)	Primary composite outcome			Cardiovascular death			Myocardial infarction			Ischemic stroke			Hemorrhagic stroke		
	HR (95% CI)^a^		−2 LL^b^	HR (95% CI)^a^		−2 LL^b^	HR (95% CI)^a^		−2 LL^b^	HR (95% CI)^a^		−2 LL^b^	HR (95% CI)^a^		−2 LL^b^
≥0 (no-threshold model)	1.05	(1.00–1.10)	7971.5	1.04	(0.95–1.12)	2535.0	1.08	(0.97–1.20)	1442.7	1.01	(0.92–1.10)	2183.3	0.89	(0.78–1.03)	944.2
≥40	1.05	(1.00–1.10)	7971.7	1.04	(0.95–1.12)	2535.0	1.08	(0.97–1.20)	1442.8	1.00	(0.92–1.10)	2183.3	0.88	(0.76–1.02)	943.9
≥50	1.05	(1.00–1.10)	7971.4	1.04	(0.95–1.13)	2534.9	1.08	(0.97–1.20)	1443.0	1.00	(0.92–1.10)	2183.3	0.87	(0.75–1.02)	**943.7**
≥60	1.06	(1.01–1.11)	7970.7	1.04	(0.96–1.15)	2534.6	1.09	(0.97–1.22)	1442.7	1.01	(0.92–1.12)	**2183.3**	0.86	(0.73–1.02)	943.7
≥70	1.07	(1.01–1.13)	**7970.1**	1.04	(0.97–1.18)	2533.8	1.11	(0.98–1.25)	**1442.3**	1.01	(0.90–1.13)	2183.3	0.87	(0.72–1.07)	944.8
≥80	1.07	(1.01–1.15)	7971.2	1.04	(0.98–1.23)	**2533.5**	1.11	(0.96–1.29)	1442.8	1.00	(0.87–1.15)	2183.3	0.92	(0.72–1.16)	946.2
≥90	1.07	(0.99–1.16)	7973.0	1.04	(0.96–1.28)	2533.9	1.10	(0.92–1.33)	1443.7	1.00	(0.84–1.19)	2183.3	0.96	(0.72–1.28)	946.7
≥100	1.06	(0.94–1.19)	7974.7	1.04	(0.92–1.35)	2534.6	1.06	(0.81–1.38)	1444.6	1.01	(0.79–1.28)	2183.3	0.95	(0.63–1.43)	946.7
≥110	1.07	(0.90–1.26)	7975.0	1.04	(0.90–1.50)	2534.6	1.01	(0.66–1.55)	1444.7	0.97	(0.67–1.14)	2183.3	0.88	(0.45–1.17)	946.6

*CI* confidence interval, *HR* hazard ratio, *LDL-C* low-density lipoprotein cholesterol, *LL* log-likelihood

^a^Per 10-mg/dl increase in LDL-C, adjusted for gender, age (<65 or 65≤ years), obesity (body mass index <25 or 25≤ kg/m^2^), diabetes mellitus, hsCRP (<1.0 or 1.0≤ mg/dl), TG (<150 or 150≤ mg/dl), HDL-C (<40 or 40≤ mg/dl), drug use (beta blocker, DAPT, or ACEI/ARB), disease history (MI, unstable angina, PCI, CABG, stroke, AF, malignant tumor, chronic heart failure, hypertension, chronic kidney disease), current smoking, and randomized group (pitavastatin 1 mg/day or 4 mg/day)

^b^The smallest value (indicated by bold number) is the best-fitted model

**Fig. 4:**
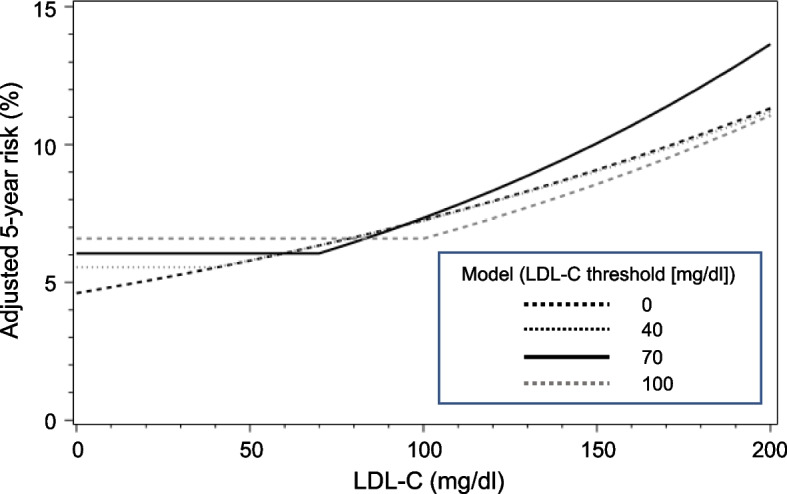
Standardized 5-year risks under the “bottoming-out” Cox models with different thresholds. The risks at each LDL-C level were predicted by the fitted model and averaged among total patients (*n* = 11,105)

The above models with distinct threshold LDL-C values were checked by residual analysis. We examined correlations between each covariate and its Schoenfeld residuals to check the proportional hazards assumption (Additional file 3: Table S3) and cumulative martingale residual plot to check the semi-linear log-hazard increase with LDL-C in the models (Additional file 4: Fig. S1). No small *p*-values were observed in any case, indicating no significant deviation from the assumptions. Figure 4 indicates the concept of these models for the primary composite outcomes, where the horizontal axis shows LDL-C and the vertical axis adjusted 5-year risk. In the black rough dotted line, where the threshold LDL-C is assumed to be 0 mg/dl, the monotonic relationship is established, representing “The lower, the better.” In the other lines, where the threshold LDL-C is assumed to be 40, 70, or 100 mg/dl, there are inflection points, and the monotonic relationship is not established in the part where LDL-C is lower than the inflection point. The black solid line demonstrates the best model fit by log-likelihood, shown for the case of a threshold LDL-C value of 70 mg/dl.

## Discussion

In this post hoc analysis of the REAL-CAD study, using a novel method named the “bottoming-out model,” we found a “threshold” value of LDL-C, below which further reduction did not affect the onset of cardiovascular events in patients with CAD given statins for secondary prevention of cardiovascular disease. Our analysis model suggests a threshold value to be 70 mg/dl for primary composite outcomes, 80 mg/dl for cardiovascular death, 70 mg/dl for myocardial infarction, and 60 mg/dl for ischemic stroke. From the results of our analysis, we can envision that the “The lower, the better” hypothesis does not always apply to Japanese CAD patients.

In the subanalysis as a preliminary analysis, to estimate the existence of the “threshold” value of LDL-C, we divided the patients into 6 categories by LDL-C level at 6 months and calculated event rates and multivariable-adjusted hazard ratios within each category. Regarding primary composite outcomes, the event rates and adjusted hazard ratios were the lowest in the 50 ≤ LDL-C < 75 mg/dl category in overall patients as well as in pitavastatin 4 mg/day group patients. The result suggested that there might be the threshold value of LDL-C around 50–75 mg/dl. Also, for other endpoints, we estimated the threshold values in the same way. Next, we performed a “bottoming-out model,” i.e., we calculated multivariable-adjusted hazard ratios for each of the threshold LDL-C values set by every 10 mg/dl. We tried several models with different threshold values of LDL-C. In the fitting of multiple models, the fitting was certainly improved in the model with the threshold set. Regarding primary composite outcomes, the hazard ratio was significant and the model fitting was best when the threshold LDL-C was assumed to be 70 mg/dl. Similarly, regarding secondary endpoints, the best-fit threshold LDL-C values were 80 mg/dl for cardiovascular death, 70 mg/dl for non-fatal myocardial infarction, and 60 mg/dl for non-fatal ischemic stroke. Our analysis model is based on the establishment of a monotonic relationship [21]. Figure 4 is a simple illustration of the bottoming-out model on the absolute risk scale. From the results of this analysis, it appears that the bottom of LDL-C is at about 70 mg/dl, as demonstrated by the black solid line in the figure. The line indicates that when LDL-C is below 70 mg/dl, the risk of cardiovascular events is constant, and when it is above 70 mg/dl, the risk increases as LDL-C rises. The uniqueness of our model is that when LDL-C is lowered below 70 mg/dl, the risk remains independent of the LDL-C level despite further reduction. These analyses were performed based on LDL-C levels at 6 months but not last LDL-C levels during the follow-up period. Since the LDL-C control continued well at the final visit as well as at 6 months in overall patients (Fig. 2C, D) and did not deteriorate before the events even in patients who had developed cardiovascular events (Fig. 3), we believe it would be rational that our analyses were conducted based on the LDL-C levels at 6 months.

The “The lower, the better” concept for LDL-C has been proposed by many statin trials [15, 22–25] and was strengthened by the recent non-statin lipid-lowering therapy trials [16–18]. The 2019 European Society of Cardiology and European Atherosclerosis Society guidelines for the management of dyslipidemias recommend aggressive goals for LDL-C lowering, such as <1.8 mmol/l (<70 mg/dl) for patients at high risk of atherosclerotic cardiovascular disease, <1.4 mmol/l (<55 mg/dl) for patients at very high risk or with clinically evident atherosclerotic cardiovascular disease, and <1.0 mmol/l (<40 mg/dl) for very high-risk patients who experienced a secondary vascular event within 2 years [26]. Some believers in the “The lower, the better” concept, especially the followers of PCSK9 inhibitors, state that no level of LDL-C below which benefit ceases or harm occurs has been defined. On the other hand, the 2018 American College of Cardiology/American Heart Association guidelines identified a threshold LDL-C value of 70 mg/dl, at which addition of a non-statin to high-intensity statin therapy could be considered, but recommends high-intensity statin therapy for the secondary prevention and for patients with high risk of atherosclerotic cardiovascular disease regardless of the baseline LDL-C without setting a specific target LDL-C, i.e., “Fire and forget,” which is the opposite concept of the “Treat to target” [27]. Therefore, regarding statin treatment, no consensus has yet reached as to “The lower, the better,” “Fire and forget,” or “Treat to target,” which has been in debate for a long time. Moreover, the universal target LDL-C for lipid-lowering treatments has not been established. In the present subanalysis, we assumed a threshold value of LDL-C every 10 mg/dl, and 70 mg/dl LDL-C showed the best-fit as the threshold value for the primary composite outcomes. The novelty of the present subanalysis lies in an aggressive search for the “bottom” of LDL-C value, i.e., the value below which events may not decrease even if it is reduced further. We believe that our “bottoming-out model” would have a certain impact, in which the shape of the assumed model was changed including the threshold value, and the model fit was compared.

Very recently, a meta-analysis result of 21 randomized clinical trials that examined the efficacy of statins on primary and secondary prevention for death and cardiovascular outcomes demonstrated was published [28]. This article demonstrated that the absolute risk reduction of treatment with statins was 0.8% (95% CI, 0.4–1.2%) for all-cause mortality, 1.3% (95% CI, 0.9–1.7%) for myocardial infarction, and 0.4% (95% CI, 0.2–0.6%) for stroke, while the relative risk reductions was 9% (95% CI, 5–14%), 29% (95% CI, 22–34%), and 14% (95% CI, 5–22%) respectively. The authors conclude that the absolute risk reductions of treatment with statins are modest compared with the relative risk reductions. From this article, the association between LDL-C levels and cardiovascular events seems modest as far as the view of absolute risk reduction is concerned, and the authors state that a conclusive association between absolute reductions in LDL-C levels and individual clinical outcomes seems pending and that discussing absolute risk reductions would be important when making informed clinical decisions with individual patients. In addition, this article seems to warn us regarding the overtreatment of patients with low baseline LDL-C levels. For that as well, we believe it is valuable that we found the threshold value of LDL-C in the present subanalysis.

The primary mechanism of statins for the prevention of cardiovascular events depends on lowering LDL-C. On the other hand, it has been proposed that statins also exert cardiovascular protective effects that are independent of LDL-C called pleiotropic effects [29, 30]. Among various pleiotropic effects, the anti-inflammatory properties of statins have been the focus of a number of clinical trials. The Myocardial Ischemia Reduction With Aggressive Cholesterol Lowering (MIRACL) trial was an acute coronary syndrome trial comparing atorvastatin 80 mg/day with placebo. Atorvastatin lowered the primary endpoint in patients with both high and normal LDL-C and also lowered the level of hsCRP by 83% [31]. JUPITER was a primary prevention trial of rosuvastatin in patients with LDL-C levels < 130 mg/dl and CRP ≥ 0.2 mg/dl. The main analysis of JUPITER found that rosuvastatin reduced LDL-C by 50%, hsCRP level by 37%, and the primary endpoint by 44% [32]. Plotting the expected benefit from JUPITER based on LDL-C lowering on the Cholesterol Treatment Trialists’ (CTT) collaboration regression line suggests that the achieved benefit may be greater than the expected benefit based on LDL-C reduction alone [25]. These observations suggest rationales for statin therapy targeting inflammation. The Canakinumab Antiinflammatory Thrombosis Outcome Study (CANTOS), which was a non-statin intervention trial for CAD, demonstrated that anti-inflammatory therapy targeting the interleukin-1β innate immunity pathway with canakinumab at a dose of 150 mg every 3 months led to a significantly lower rate of recurrent cardiovascular events than placebo, independent of LDL-C lowering [33]. Therefore, anti-inflammatory interventions as well as LDL-C lowering might be beneficial in reducing cardiovascular events in patients with CAD. In the present subanalysis, we demonstrated that a threshold value of LDL-C was present and that it was 70 mg/dl for the primary composite outcome. From our results, we can envision that therapy targeting residual risk factors beyond LDL-C, including inflammation, would be promising, for secondary prevention in CAD patients with LDL-C < 70 mg/dl. Also, in the REAL-CAD study, another subanalysis focused on hsCRP levels is currently in progress.

### Study limitations

In this subanalysis, we used a novel analysis procedure called the “bottoming-out model” to assess the monotonic between LDL-C levels and cardiovascular event onset. There are some points to keep in mind in this method. The thresholds of LDL-C relationship around 70–90 mg/dl obtained from our analysis also rely on the modeling assumptions of proportional hazards, that is, time-constant hazard ratios and the linear association with hazard for each adjustment variable. Although we checked these assumptions in primary models via the Schoenfeld (for proportional hazards assumption) and martingale (for linearity assumption) residuals and did not find evidence for gross deviation (Additional file 4: Fig. S1 and Additional file 3: Table S3), partly linear relationship with a single threshold characterized by our “bottoming-out” models is itself a strong assumption. Hence, the results should be taken as a simplified approximation of the possibly non-monotonic associations between LDL-C and cardiovascular risks. In the first place, the “bottoming-out model” did not attempt to clarify the “threshold” with a statistical significance. Although we found from the shape of model fitting that the “threshold” might exist, the value (negative double of the log-likelihood or −2LL) of the model fitting does not have criteria to interpret how much the difference is significant. Therefore, it might be impossible to make a strong claim that there is the definitive “threshold,” only from our results. The bottoming-out model (Table 3, Fig. 4) directly incorporates a hypothesized piecewise log-linear association between LDL-C levels and cardiovascular events with specific thresholds, where the lower LDL-C level is not associated with an increased risk of cardiovascular events. The advantage of the model would be the simple, parsimonious generalization of the strictly increasing log-linear association typically assumed in the standard Cox models. However, our model also relies on the abovementioned restricted piecewise log-linear relationship. If the model severely misspecifies the true hazard changes by LDL-C levels, the results from the bottoming-out model would be misleading as with other statistical modeling techniques.

The REAL-CAD trial is the largest scale trial in the world. However, it is a study of Japanese CAD patients alone and does not take into account racial differences. Primarily, cardiovascular risk is lower, and cardiovascular event rates are lower in Japanese CAD patients, compared with Western patients. In also this subanalysis, the number of cardiovascular events was too small, especially in patients with LDL-C <70 mg/dl, and was still smaller in patients with LDL-C <50 mg/dl. It is undeniable that these facts affected the conclusions obtained from this subanalysis. Although we have discussed the inflammatory response as a residual risk beyond LDL-C, hsCRP levels in Japanese CAD patients are lower than those in Western patients. Therefore, detailed investigations for racial differences are needed. We hope a similar analysis to the present one will be applied also to the Western CAD patients. Finally, in the REAL-CAD trial, the overall population was only followed for 5 years and some cardiovascular events may not have been fully exposed. In most of the large clinical trials, long-term results have been observed for 2, 3, or 5 years at most. Still longer-term follow-up would be necessary when considering the life span of a person, although it may be very difficult.

### Clinical implications

PCSK9 inhibitors, when added to a statin, can achieve an LDL-C level as near to zero as possible [17, 18]. Estimating from the CTT collaboration regression line, theoretically, the incidence of cardiovascular events would be zero, if the LDL-C level were under 30 mg/dl in patients with CAD [25]. Actually, however, cardiovascular events may occur even if the LDL-C level is near to zero. We reported a case of a 76-year-old woman with CAD who experienced several repeated cardiovascular events after PCI, despite receiving a PCSK9 inhibitor, evolocumab (420 mg every month), along with a moderate dose of a statin (5.0 mg/day rosuvastatin) and that her LDL-C was as low as 10 mg/dl. However, finally by increasing the dose of rosuvastatin to 20 mg/day (the maximum in Japan), her hsCRP level was reduced from 2.4 to 0.09 mg/l, after which she no longer developed cardiovascular events [19]. In the main results of the REAL-CAD trial, the pitavastatin 4 mg/day group achieved a significant reduction of hsCRP at 6 months from baseline at randomization, while pitavastatin 1 mg/day showed no change in hsCRP, possibly explaining in part the greater reduction of cardiovascular events in the pitavastatin 4 mg/day (maximum dose in Japan) group. In the present subanalysis, we first estimated that a “threshold” value of LDL-C might exist around 50–75 mg/dl for primary composite outcomes, especially in patients receiving high-dose statin that suppressed inflammatory reaction more strongly. Next, following “bottoming-out model” exhibited that the “threshold” value of LDL-C might be 70 mg/dl. These results suggest that the “The lower, the better” concept applies until LDL-C is reduced to 70 mg/dl, but not when the level is below 70 mg/dl. Therefore, our analysis model provided us a certain proposal that we should aim “Treat to target” until lowering LDL-C to less than 70 mg/dl. When the LDL-C level reaches less than 70 mg/dl, we should then use high-dose statins with the concept “Fire and forget.” Taken together, we can envision a lipid-lowering strategy for secondary prevention of CAD as follows: First, we aim to reduce LDL-C to less than 70 mg/dl and also to reduce inflammatory status, by increasing the dose of statins to the maximum. Next, if the LDL-C level does not reach 70 mg/dl even after using the maximum dose of the statins, a non-statin drug such as ezetimibe or PCSK9 inhibitors is used in combination.

## Conclusions

When statins were given for secondary prevention of cardiovascular events in Japanese patients with CAD, a “threshold” value of LDL-C, below which further lowering did not affect the onset of cardiovascular events, might be present. That is, the risk of cardiovascular events might decrease monotonically until LDL-C was lowered to the threshold, but that even when LDL-C was lowered below the threshold, the risk might remain independent of the LDL-C level. Such a threshold value might be 70 mg/dl for composite cardiovascular outcomes. This result might provide us a novel lipid-lowering strategy for the secondary prevention of CAD in Japan, i.e., “Treat to target” to 70 mg/dl and thereafter “Fire and forget.”

## Supplementary Information

**Additional file 1: Table S1.** LDL-C category-specific event rates (pitavastatin 4 mg/day group). Data are adjusted for gender, age (<65 or 65≤ years), obesity (body mass index <25 or 25≤ kg/m2), diabetes mellitus, hsCRP (<1.0 or 1.0≤ mg/dl), TG (<150 or 150≤ mg/dl), HDL-C (<40 or 40≤ mg/dl), drug use (beta blockers, dual antiplatelet therapy, or ACE inhibitors/ARBs), disease history (myocardial infarction, unstable angina, PCI, CABG, stroke, atrial fibrillation, malignant tumor, chronic heart failure, hypertension, chronic kidney disease) and current smoking. Adjusted HR and 95% CI in each category are shown as the values when the category 100≤ LDL-C <125 was used as a reference.

**Additional file 2: Table S2.** LDL-C category-specific event rates (pitavastatin 1 mg/day group). Data are adjusted for gender, age (<65 or 65≤ years), obesity (body mass index <25 or 25≤ kg/m2), diabetes mellitus, hsCRP (<1.0 or 1.0≤ mg/dl), TG (<150 or 150≤ mg/dl), HDL-C (<40 or 40≤ mg/dl), drug use (beta blockers, dual antiplatelet therapy, or ACE inhibitors/ARBs), disease history (myocardial infarction, unstable angina, PCI, CABG, stroke, atrial fibrillation, malignant tumor, chronic heart failure, hypertension, chronic kidney disease) and current smoking. Adjusted HR and 95% CI in each category are shown as the values when the category 100≤ LDL-C <125 was used as a reference.

**Additional file 3: Table S3.** Assessment for the deviation from time-constant assumption for hazard ratios. Asterisks exhibit correlation between the Schoenfeld residuals and rank-transformed time at events. Daggers mean that *P* value is based on Chi-square test for time-constant hazard ratio.

**Additional file 4: Figure S1.** The cumulative martingale residuals (blue, thick line) plotted against 10-mg/dl LDL-C levels above the threshold value set by each model to check the linearity assumption in the Cox models, accompanied by 1000 resampled zero-mean random Gaussian processes (black, thin lines). If the relationship between LDL-C above the threshold and event hazards is actually linear in the Cox model (i.e., the linearity assumption holds), the cumulative sum of martingale residuals will approximately follow the zero-mean Gaussian process and fluctuate randomly. The supremum of the cumulative martingale residuals along the LDL-C levels can be tested against the suprema from randomly sampled zero-mean Gaussian processes. A small p-value from this resampling-based test would provide evidence against the linearity assumption.

## Data Availability

The dataset used in this study may be requested from the Public Health Research Foundation for reasonable grounds. However, the data, analytical methods, and study materials will not be made available to other researchers for purposes of reproducing the results or replicating the procedure.

## Abbreviations

ACE: Angiotensin-converting enzyme
ARBs: Angiotensin receptor blockers
ARC: Academic Research Consortium
CABG: Coronary artery bypass surgery
CAD: Coronary artery disease
CANTOS: Canakinumab Antiinflammatory Thrombosis Outcome Study
CTT: Cholesterol Treatment Trialists
HDL-C: High-density lipoprotein cholesterol
HMG-CoA: 3-Hydroxy-3-methylglutaryl coenzyme A
hsCRP: High-sensitivity C-reactive protein
JUPITER: Justification for the Use of the Statins in Prevention an Intervention Trial Evaluating Rosuvastatin
LDL-C: Low-density lipoprotein cholesterol
MIRACL: Myocardial Ischemia Reduction With Aggressive Cholesterol Lowering
PCI: Percutaneous coronary intervention
PCSK9: Proprotein convertase subtilisin/kexin type 9
REAL-CAD: Randomized Evaluation of Aggressive or Moderate Lipid Lowering Therapy With Pitavastatin in Coronary Artery Disease
TG: Triglyceride
