# Supplementary material for: Optimal target of LDL cholesterol level for statin treatment: challenges to monotonic relationship with cardiovascular events
Source: BMC Med. 2022 Nov 14;20:441. doi: 10.1186/s12916-022-02633-5 (PMC9661797; doi:10.1186/s12916-022-02633-5)
Supplement: Supplementary file 1 — Additional file 1: Table S1. LDL-C category-specific event rates (pitavastatin 4 mg/day group). Data are adjusted for gender, age (<65 or 65≤ years), obesity (body mass index <25 or 25≤ kg/m2), diabetes mellitus, hsCRP (<1.0 or 1.0≤ mg/dl), TG (<150 or 150≤ mg/dl), HDL-C (<40 or 40≤ mg/dl), drug use (beta blockers, dual antiplatelet therapy, or ACE inhibitors/ARBs), disease history (myocardial infarction, unstable angina, PCI, CABG, stroke, atrial fibrillation, malignant tumor, chronic heart failure, hypertension, chronic kidney disease) and current smoking. Adjusted HR and 95% CI in each category are shown as the values when the category 100≤ LDL-C <125 was used as a reference. [file 12916_2022_2633_MOESM1_ESM.docx]

| **Table S1**  LDL-C category-specific event rates (pitavastatin 4 mg/day group) | | | | | | | |
| --- | --- | --- | --- | --- | --- | --- | --- |
|  |  | Achieved on-trial LDL-C level (mg/dl) | | | | | |
|  |  | LDL-C <50 | 50≤ LDL-C <75 | 75≤ LDL-C <100 | 100≤ LDL-C <125 | 125≤ LDL-C <150 | 150≤ LDL-C |
| Endpoint | | *n* = 507 | *n* = 2544 | *n* = 1904 | *n* = 346 | *n* = 39 | *n* = 6 |
| Primary composite outcome, *n* | | 28 | 91 | 73 | 30 | 2 | 1 |
|  | Rate (/1000 person-years) | 14.9 | 9.5 | 10.2 | 24.5 | 15.1 | 47.4 |
|  | Adjusted HR (95% CI) | 0.44 (0.25, 0.77) | 0.30 (0.19, 0.47) | 0.32 (0.20, 0.51) | Ref | 0.67 (0.16, 2.85) | 1.63 (0.22, 12.29) |
| Cardiovascular death, *n* | | 10 | 33 | 17 | 9 | 0 | 1 |
|  | Rate (/1000 person-years) | 5.2 | 3.4 | 2.3 | 7.1 | - | 47.4 |
|  | Adjusted HR (95% CI) | 0.52 (0.18, 1.48) | 0.41 (0.18, 0.97) | 0.33 (0.13, 0.80) | Ref | - | 6.05 (0.68, 53.74) |
| Myocardial infarction, *n* | | 4 | 13 | 13 | 5 | 1 | 0 |
|  | Rate (/1000 person-years) | 2.1 | 1.3 | 1.8 | 4.0 | 7.3 | - |
|  | Adjusted HR (95% CI) | 0.28 (0.07, 1.38) | 0.18 (0.06, 0.59) | 0.25 (0.08, 0.83) | Ref | 2.11 (0.22, 19.81) | - |
| Ischemic stroke, *n* | | 9 | 28 | 23 | 8 | 0 | 0 |
|  | Rate (/1000 person-years) | 4.7 | 2.9 | 3.2 | 6.4 | - | - |
|  | Adjusted HR (95% CI) | 0.50 (0.18, 1.38) | 0.31 (0.13, 0.74) | 0.30 (0.12, 0.75) | Ref | - | - |
| Hemorrhagic stroke, *n* | | 3 | 20 | 7 | 3 | -0 | 0 |
|  | Rate (/1000 person-years) | 1.6 | 2.1 | 1.0 | 2.4 | - | - |
|  | Adjusted HR (95% CI) | 0.96 (0.15, 5.97) | 1.31 (0.30, 5.82) | 0.45 (0.09, 2.36) | Ref | - | - |
| CI, confidence interval; HR, hazard ratio; LDL-C, low-density lipoprotein cholesterol | | | | |  |  |  |
| * Adjusted for gender, age (<65 or 65≤ years), obesity (body mass index <25 or 25≤ kg/m^2^), diabetes mellitus, hsCRP (<1.0 or 1.0≤ mg/dl), TG (<150 or 150≤   mg/dl), HDL-C (<40 or 40≤ mg/dl), drug use (beta blockers, dual antiplatelet therapy, or ACE inhibitors/ARBs), disease history (myocardial infarction, unstable  angina, PCI, CABG, stroke, atrial fibrillation, malignant tumor, chronic heart failure, hypertension, chronic kidney disease) and current smoking. | | | | | | | |
| * Adjusted HR and 95% CI in each category are shown as the values when the category 100≤ LDL-C <125 was used as a reference. | | | | | | | |
