# Supplementary material for: Optimal target of LDL cholesterol level for statin treatment: challenges to monotonic relationship with cardiovascular events
Source: BMC Med. 2022 Nov 14;20:441. doi: 10.1186/s12916-022-02633-5 (PMC9661797; doi:10.1186/s12916-022-02633-5)
Supplement: Supplementary file 2 — Additional file 2: Table S2. LDL-C category-specific event rates (pitavastatin 1 mg/day group). Data are adjusted for gender, age (<65 or 65≤ years), obesity (body mass index <25 or 25≤ kg/m2), diabetes mellitus, hsCRP (<1.0 or 1.0≤ mg/dl), TG (<150 or 150≤ mg/dl), HDL-C (<40 or 40≤ mg/dl), drug use (beta blockers, dual antiplatelet therapy, or ACE inhibitors/ARBs), disease history (myocardial infarction, unstable angina, PCI, CABG, stroke, atrial fibrillation, malignant tumor, chronic heart failure, hypertension, chronic kidney disease) and current smoking. Adjusted HR and 95% CI in each category are shown as the values when the category 100≤ LDL-C <125 was used as a reference. [file 12916_2022_2633_MOESM2_ESM.docx]

| **Table S2**  LDL-C category-specific event rates (pitavastatin 1 mg/day group) | | | | | | | |
| --- | --- | --- | --- | --- | --- | --- | --- |
|  |  | Achieved on-trial LDL-C level (mg/dl) | | | | | |
|  |  | LDL-C <50 | 50≤ LDL-C <75 | 75≤ LDL-C <100 | 100≤ LDL-C <125 | 125≤ LDL-C <150 | 150≤ LDL-C |
| Endpoint | | *n* = 152 | *n* = 1287 | *n* = 2655 | *n* = 1719 | *n* = 1373 | *n* = 8 |
| Primary composite outcome, *n* | | 4 | 66 | 157 | 67 | 13 | 1 |
|  | Rate (/1000 person-years) | 13.1 | 10.9 | 13.6 | 15.3 | 13.5 | 18.6 |
|  | Adjusted HR (95% CI) | 0.39 (0.12, 1.24) | 0.96 (0.66, 1.39) | 1.24 (0.91, 1.69) | Ref | 1.21 (0.65, 2.27) | 0.88 (0.12, 6.43) |
| Cardiovascular death, *n* | | 2 | 25 | 47 | 19 | 7 | 0 |
|  | Rate (/1000 person-years) | 3.5 | 5.1 | 4.7 | 3.6 | 7.1 | - |
|  | Adjusted HR (95% CI) | 0.78 (0.12, 1.24) | 1.11 (0.58, 2.14) | 1.22 (0.68, 2.19) | Ref | 2.33 (0.90, 5.99) | - |
| Myocardial infarction, *n* | | 1 | 12 | 14 | 18 | 2 | 0 |
|  | Rate (/1000 person-years) | 1.8 | 2.5 | 3.4 | 3.5 | 2.0 | - |
|  | Adjusted HR (95% CI) | - | 0.63 (0.29, 1.41) | 0.91 (0.49, 1.68) | Ref | 0.75 (0.17, 3.28) | - |
| Ischemic stroke, *n* | | 1 | 19 | 37 | 13 | 4 | 0 |
|  | Rate (/1000 person-years) | 1.8 | 3.9 | 3.7 | 2.5 | 4.1 | - |
|  | Adjusted HR (95% CI) | 0.60 (0.08, 4.65) | 1.43 (0.67, 3.06) | 1.56 (0.79, 3.09) | Ref | 1.89 (0.60, 5.98) | - |
| Hemorrhagic stroke, *n* | | 0 | 14 | 6 | 8 | 2 | 0 |
|  | Rate (/1000 person-years) | - | 2.9 | 0.6 | 1.5 | 2.0 | - |
|  | Adjusted HR (95% CI) | - | 2.30 (0.85, 6.22) | 0.50 (0.16, 1.56) | Ref | 0.90 (0.11, 7.67) | - |
| CI, confidence interval; HR, hazard ratio; LDL-C, low-density lipoprotein cholesterol | | | | |  |  |  |
| * Adjusted for gender, age (<65 or 65≤ years), obesity (body mass index <25 or 25≤ kg/m^2^), diabetes mellitus, hsCRP (<1.0 or 1.0≤ mg/dl), TG (<150 or 150≤   mg/dl), HDL-C (<40 or 40≤ mg/dl), drug use (beta blockers, dual antiplatelet therapy, or ACE inhibitors/ARBs), disease history (myocardial infarction, unstable  angina, PCI, CABG, stroke, atrial fibrillation, malignant tumor, chronic heart failure, hypertension, chronic kidney disease) and current smoking. | | | | | | | |
| * Adjusted HR and 95% CI in each category are shown as the values when the category 100≤ LDL-C <125 was used as a reference. | | | | | | | |
