# Supplementary material for: Optimal target of LDL cholesterol level for statin treatment: challenges to monotonic relationship with cardiovascular events
Source: BMC Med. 2022 Nov 14;20:441. doi: 10.1186/s12916-022-02633-5 (PMC9661797; doi:10.1186/s12916-022-02633-5)
Supplement: Supplementary file 3 — Additional file 3: Table S3. Assessment for the deviation from time-constant assumption for hazard ratios. Asterisks exhibit correlation between the Schoenfeld residuals and rank-transformed time at events. Daggers mean that P value is based on Chi-square test for time-constant hazard ratio. [file 12916_2022_2633_MOESM3_ESM.docx]

| **Table S3** Assessment for the deviation from time-constant assumption for hazard ratios. | | | | | | | | | | | |
| --- | --- | --- | --- | --- | --- | --- | --- | --- | --- | --- | --- |
|  | Model (LDL-C threshold) | | | | | | | | | | |
|  | Model 1 (0 mg/dl) | |  | Model 2 (40 mg/dl) | |  | Model 3 (70 mg/dl) | |  | Model 4 (100 mg/dl) | |
|  | Corr (Residual, time)* | P† |  | Corr (Residual, time)* | P† |  | Corr (Residual, time)* | P† |  | Corr (Residual, time)* | P† |
| **LDL-C (mg) above threshold** | 0.0779 | 0.0895 |  | 0.0795 | 0.0809 |  | 0.0575 | 0.2226 |  | 0.0523 | 0.2591 |
| Age ≥65 | -0.0807 | 0.0907 |  | -0.0804 | 0.0916 |  | -0.0818 | 0.0858 |  | -0.0824 | 0.083 |
| Male gender | 0.0229 | 0.6271 |  | 0.0231 | 0.624 |  | 0.0208 | 0.6589 |  | 0.0189 | 0.6869 |
| Body mass index ≥25 | -0.0691 | 0.1492 |  | -0.069 | 0.1499 |  | -0.0687 | 0.1516 |  | -0.0684 | 0.1537 |
| Diabetes mellitus | 0.0788 | 0.0982 |  | 0.0789 | 0.0977 |  | 0.0777 | 0.1031 |  | 0.0773 | 0.1049 |
| hsCRP (6 month) ≥1 mg/dl | 0.00844 | 0.8563 |  | 0.00854 | 0.8547 |  | 0.00869 | 0.8521 |  | 0.00744 | 0.8733 |
| TG ≥150 mg/dl | -0.00845 | 0.8573 |  | -0.00861 | 0.8546 |  | -0.0103 | 0.8258 |  | -0.00985 | 0.8338 |
| HDL-C ≥40 mg/dl | -0.0178 | 0.7014 |  | -0.0176 | 0.7051 |  | -0.0194 | 0.676 |  | -0.0211 | 0.65 |
| Beta blockers | 0.044 | 0.3434 |  | 0.044 | 0.3437 |  | 0.0442 | 0.342 |  | 0.0452 | 0.3306 |
| Dual anti-platelet therapy | -0.0434 | 0.3555 |  | -0.0435 | 0.3541 |  | -0.0429 | 0.3616 |  | -0.0423 | 0.3685 |
| ACE inhibitors/ARBs | 0.051 | 0.2694 |  | 0.051 | 0.2697 |  | 0.0504 | 0.2744 |  | 0.05 | 0.2791 |
| Previous myocardial infarction | -0.00042 | 0.9927 |  | -0.00056 | 0.9904 |  | 0.00112 | 0.9806 |  | 0.00241 | 0.9584 |
| Previous unstable angina | -0.0271 | 0.5612 |  | -0.0271 | 0.5601 |  | -0.0263 | 0.572 |  | -0.0253 | 0.5867 |
| Previous PCI | -0.0274 | 0.5557 |  | -0.0274 | 0.5555 |  | -0.0276 | 0.5525 |  | -0.0269 | 0.5621 |
| Previous CABG | 0.0868 | 0.0565 |  | 0.0867 | 0.0567 |  | 0.0871 | 0.0558 |  | 0.0863 | 0.0577 |
| Previous stroke | 0.0172 | 0.7146 |  | 0.0172 | 0.7142 |  | 0.0163 | 0.7285 |  | 0.0148 | 0.7522 |
| Previous atrial fibrillation | 0.0609 | 0.1918 |  | 0.061 | 0.191 |  | 0.0604 | 0.1956 |  | 0.0585 | 0.2088 |
| Previous malignant tumor | 0.0226 | 0.6293 |  | 0.0227 | 0.6279 |  | 0.0225 | 0.6318 |  | 0.0223 | 0.6348 |
| Previous chronic heart failure | 0.00626 | 0.8932 |  | 0.0062 | 0.8942 |  | 0.00609 | 0.8961 |  | 0.00579 | 0.9011 |
| Hypertension | 0.0595 | 0.2104 |  | 0.0597 | 0.2092 |  | 0.0591 | 0.2133 |  | 0.0582 | 0.2208 |
| Chronic kidney disease (stage 3, 4, 5) | 0.0107 | 0.8205 |  | 0.0106 | 0.8218 |  | 0.00956 | 0.8389 |  | 0.00825 | 0.8606 |
| Smoker | -0.0184 | 0.6916 |  | -0.0186 | 0.6891 |  | -0.0189 | 0.6848 |  | -0.0176 | 0.7046 |
| Randomized group (4 mg) | 0.0318 | 0.4863 |  | 0.0326 | 0.475 |  | 0.0223 | 0.621 |  | 0.0124 | 0.7883 |
| * Correlation between the Schoenfeld residuals and rank-transformed time at events. | | | | |  |  |  |  |  |  |  |
| † Chi-square test for time-constant hazard ratio. | |  |  |  |  |  |  |  |  |  |  |
