# Supplementary material for: Optimal target of LDL cholesterol level for statin treatment: challenges to monotonic relationship with cardiovascular events
Source: BMC Med. 2022 Nov 14;20:441. doi: 10.1186/s12916-022-02633-5 (PMC9661797; doi:10.1186/s12916-022-02633-5)
Supplement: Supplementary file 4 — Additional file 4: Figure S1. The cumulative martingale residuals (blue, thick line) plotted against 10-mg/dl LDL-C levels above the threshold value set by each model to check the linearity assumption in the Cox models, accompanied by 1000 resampled zero-mean random Gaussian processes (black, thin lines). If the relationship between LDL-C above the threshold and event hazards is actually linear in the Cox model (i.e., the linearity assumption holds), the cumulative sum of martingale residuals will approximately follow the zero-mean Gaussian process and fluctuate randomly. The supremum of the cumulative martingale residuals along the LDL-C levels can be tested against the suprema from randomly sampled zero-mean Gaussian processes. A small p-value from this resampling-based test would provide evidence against the linearity assumption. [file 12916_2022_2633_MOESM4_ESM.pptx]

## Slide 1
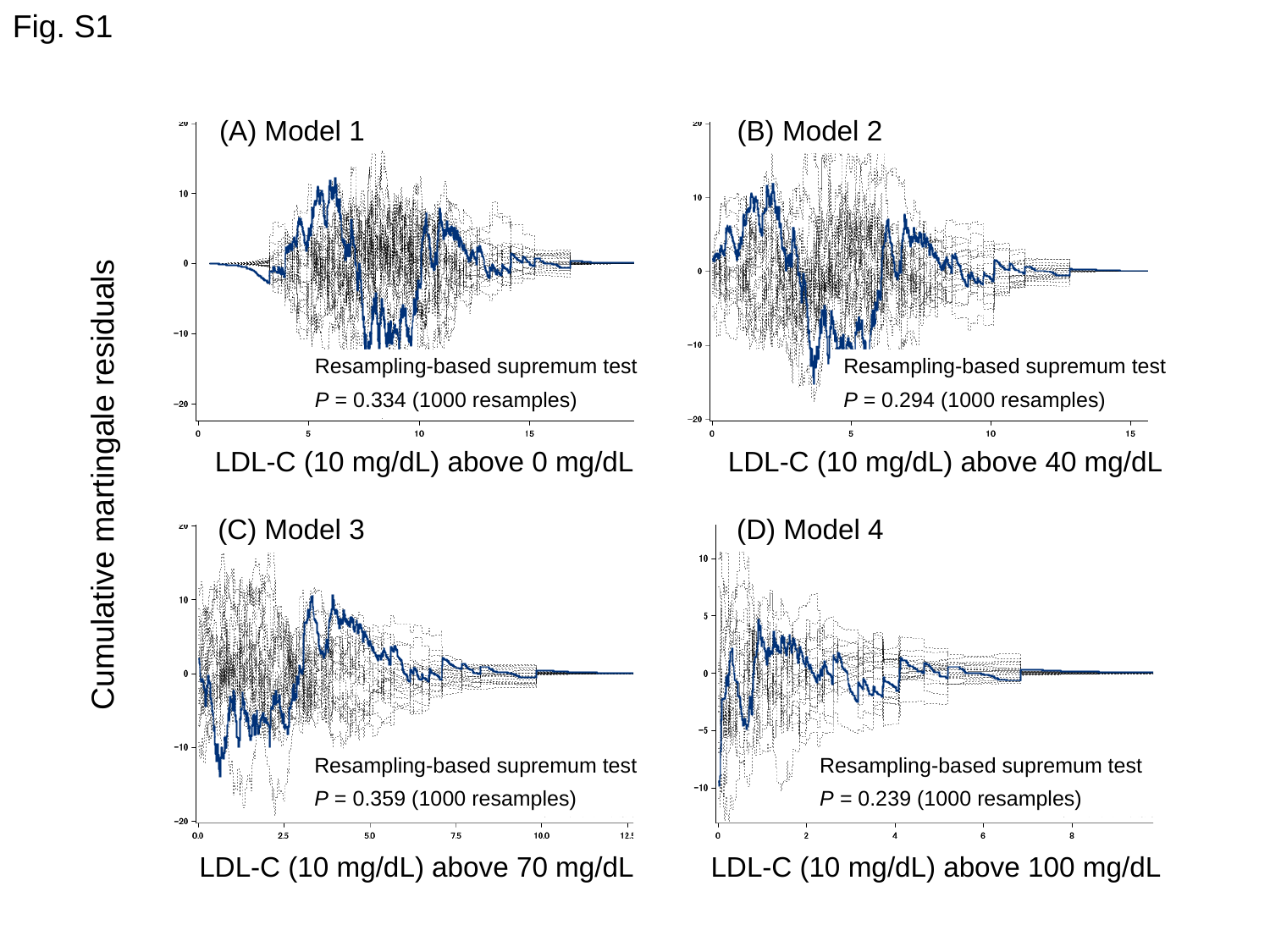

Fig. S1
(A) Model 1
(B) Model 2
Resampling-based supremum test
P = 0.334 (1000 resamples)
LDL-C (10 mg/dL) above 0 mg/dL
Resampling-based supremum test
P = 0.294 (1000 resamples)
LDL-C (10 mg/dL) above 40 mg/dL
Cumulative martingale residuals
(C) Model 3
 (D) Model 4
Resampling-based supremum test
P = 0.359 (1000 resamples)
 LDL-C (10 mg/dL) above 70 mg/dL
Resampling-based supremum test
P = 0.239 (1000 resamples)
 LDL-C (10 mg/dL) above 100 mg/dL
